# PAH–DNA Adducts in Cord Blood and Fetal and Child Development in a Chinese Cohort

**DOI:** 10.1289/ehp.8939

**Published:** 2006-04-04

**Authors:** Deliang Tang, Tin-yu Li, Jason J. Liu, Yu-hui Chen, Lirong Qu, Frederica Perera

**Affiliations:** 1 Department of Environmental Health Sciences and; 2 Columbia Center for Children’s Environmental Health, Mailman School of Public Health, Columbia University, New York, New York, USA; 3 Chongqing Children Hospital, Chongqing, China

**Keywords:** birth outcome, coal-burning emission, cord blood, fetal and child development, PAH-DNA adducts

## Abstract

Polycyclic aromatic hydrocarbons (PAHs) are an important class of toxic pollutants released by fossil fuel combustion. Other pollutants include metals and particulate matter. PAH–DNA adducts, or benzo[*a*]pyrene (BaP) adducts as their proxy, provide a chemical-specific measure of individual biologically effective doses that have been associated with increased risk of cancer and adverse birth outcomes. In the present study we examined the relationship between prenatal PAH exposure and fetal and child growth and development in Tongliang, China, where a seasonally operated coal-fired power plant was the major pollution source. In a cohort of 150 nonsmoking women and their newborns enrolled between 4 March 2002 and 19 June 2002, BaP–DNA adducts were measured in maternal and umbilical cord blood obtained at delivery. The number of gestational months occurring during the period of power plant operation provided a second, more general measure of exposure to plant emissions, in terms of duration. High PAH–DNA adduct levels (above the median of detectable adduct level) were associated with decreased birth head circumference (*p* = 0.057) and reduced children’s weight at 18 months, 24 months, and 30 months of age (*p* < 0.05), after controlling for potential confounders. In addition, in separate models, longer duration of prenatal exposure was associated with reduced birth length (*p* = 0.033) and reduced children’s height at 18 (*p* = 0.001), 24 (*p* < 0.001), and 30 months of age (*p* < 0.001). The findings suggest that exposure to elevated levels of PAHs, with the Tongliang power plant being a significant source, is associated with reduced fetal and child growth in this population.

In many industrialized regions of the world, excessive exposure to air pollutants threatens the health of the fetus and the young developing child. Evidence suggests that fetuses and children are more sensitive than adults to the toxicity of many environmental air pollutants because of their higher cell proliferation rates, lower immunologic competence, and decreased ability to detoxify carcinogens and to repair DNA damage (Anderson et al. 2000; National Research Council 1993; Perera et al. 2004; World Health Organization 1986).

Polycyclic aromatic hydrocarbons (PAHs) are among the most harmful air pollutants and are generated by the incomplete combustion of fossil fuels such as coal, diesel, and gasoline. PAHs are also present in tobacco smoke and grilled or broiled foods. A number of PAHs, including benzo[*a*]pyrene (BaP), are known human mutagens and carcinogens. Some PAHs are transplacental carcinogens in experimental bioassays, producing tumors in the liver, lung, lymphatic tissues, and nervous system of the offspring (Bulay and Wattenberg 1971; Rice and Ward 1982; Vesselinovitch et al. 1975). There is growing evidence that PAHs are also developmental toxicants in humans (Dejmek et al. 2000; Perera et al. 1998, 2003, 2004; Wu et al. 2003). Because PAH–DNA adducts reflect individual variation in exposure, absorption, metabolic activation, and DNA repair, they provide an informative individual biologic dosimeter and risk marker.

Currently, the Columbia Center for Children’s Environmental Health is carrying out three parallel studies in Krakow (Poland), New York City (USA), and Tongliang (China) to examine the impact of *in utero* exposure to airborne PAHs and other combustion-related pollutants on the health and development of newborns (Perera et al. 2005). The present study in Tongliang was carried out in collaboration with the Chongqing University of Medical Sciences and the Desert Research Institute.

The city of Tongliang has a population of approximately 810,000, and the birth rate is at 3–8/1,000. The city is situated in a small basin approximately 3 km in diameter. A coal-fired power plant located south of the town center operated during the dry season from 1 December to 31 May before 2004 to compensate for the insufficient hydraulic power during that time period. This plant was the principal source of local air pollution, because in 1995 nearly all domestic heating and cooking units were converted to natural gas, and motor vehicles are not a major source. The plant was not equipped with modern pollution reduction technology and combusted about 25,000 tons of high-sulfur coal during each annual 6-month period of operation. This report concerns a cohort of newborns whose gestational period overlapped with the months of power plant operation from 1 December 2001 to 31 May 2002.

As reported previously, the air monitoring data collected as part of this study indicate that Tongliang has a higher PAH level than does Krakow or New York City (Chow et al. 2006; Perera et al. 2005). The monitoring data also suggest that marked seasonal variation in air pollution was attributable in large part to power plant emissions (Chow et al. 2006). For example, concentrations of PAHs and particulate matter with aerodynamic diameter ≤ 2.5 μm (PM_2.5_) were highest during the winter (December–February) and lowest during the summer (June–August). Concentrations of ambient PAHs of relatively high molecular weight (168–266) were 1.5–3.5 times higher during the power plant’s operational period. PAHs in this molecular weight range include BaP and benzo[*e*]pyrene, which are known byproducts of coal combustion. The air monitoring data thus provide compelling evidence that the power plant was the major contributor to PAHs in air.

We hypothesized that higher PAH biologic effective doses, as measured by higher levels of PAH–DNA adducts in the umbilical cord blood, will lead to worse birth outcomes and affect the physical growth of the children. Our previous study in Krakow showed that mothers and newborns with higher exposure to ambient air pollution had increased PAH–DNA adducts (Whyatt et al. 1998), and that newborns with more PAH–DNA adducts had significantly decreased birth weight, birth length, and head circumference (Perera et al. 1999). We also hypothesized that longer durations of *in utero* exposure would be associated with worse outcomes.

The power plant was permanently shut down in May 2004 after the Tongliang County government determined that its shutdown would significantly improve local health and have minimal adverse social and economic impacts. After the shutdown, we enrolled a second cohort of mothers and newborns to compare the health of children with and without *in utero* exposure to the emissions of the power plant. The data collected from that cohort will appear in future publications. Because coal-fired power plants currently produce 75% of China’s electricity and most new plants are being built to burn coal, results from the Tongliang study have implications for the health of many other children in China.

## Materials and Methods

### Study subjects

The subjects are 150 children born to nonsmoking Chinese women who gave birth at the Tongliang County Hospital, Tongliang Traditional Chinese Medicine Hospital, Tongliang Maternal Children Health Hospital, and Bachuan Hospital between 4 March 2002 and 19 June 2002. The women were selected using a screening questionnaire when they checked in for delivery. Eligibility criteria included current non-smoking status, ≥ 20 years of age, and residence within 2.5 km of the Tongliang power plant. We recruited every eligible woman who gave birth at one of the four hospitals between 4 March 2002 and 19 June 2002. Their demographic characteristics are presented in Table 1. All eligible women agreed to enter the study, with 10.6% (16 of 150) lost to follow-up through their children’s age of 30 months. All subjects gave informed written consent by completing a form approved by the Columbia University Institutional Review Board and Chongqing University of Medical Sciences.

### Personal interview

A 45-min questionnaire was administered by a trained interviewer after delivery. The questionnaire elicited demographic information, lifetime residential history (location of birth and duration of residence), history of active and passive smoking (including number of household members who smoke), occupational exposure, medication information, alcohol use during each trimester of pregnancy, and consumption of PAH-containing meat (frequency of eating fried, broiled, or barbecued meat during the last 2 weeks). Socioeconomic information related to income and education was also collected.

### Biologic sample collection and analysis

Maternal blood (10 mL) was collected within 1 day postpartum, and umbilical cord blood (40–60 mL) was collected at delivery. Samples were transported to the field laboratory at the Tongliang County Hospital immediately after collection. All samples were processed there. For blood samples, the buffy coat, packed red blood cells, and plasma were separated and stored at −70°C.

### DNA adducts

We analyzed BaP–DNA adducts in extracted white blood cell (WBC) DNA using the modified high-performance liquid chromatography–fluorescence method (Alexandrov et al. 1992), which detects BaP tetrols. This assay is a sensitive and specific method for measuring BaP–DNA adducts in WBCs from individuals exposed to BaP (Bartsch and Hietanen 1996) and has a 12% coefficient of variation (Perera et al. 2005). Among 150 blood samples, 19 of them did not have enough DNA for the assay. Of the remaining 131 samples, 104 had detectable adduct levels, and 27 had nondetectable adduct levels. We assigned a value of 0.125, which is half of the adduct detection limit of 0.25, to the nondetectable samples. Samples were run coded, and samples from mother–child pairs were run in the same batch to minimize batch effects.

### Measures relevant to birth outcomes and physical development

Birth weight, birth length, and head circumference were measured immediately after parturition. Information abstracted by the research workers from mothers’ and infants’ medical records after delivery included date of delivery; gestational age at birth (based on the last menstrual period); infant sex, birth weight, length, head circumference, and malformations; maternal height, prepregnancy weight, and total weight gain; complications of pregnancy and delivery; and medications used during pregnancy. After the newborns reached 18 months of age, their weight, height, and head circumference were periodically measured at 6-month intervals as indicators of physical development.

### Duration of exposure

The estimated duration of *in utero* exposure to power plant operation was based on the number of months of pregnancy that overlapped with the period of plant operation (1 December 2001 to 31 May 2002). All births occurred between 4 March 2002 and 19 June 2002. Thus, the duration of prenatal exposure to power plant emissions ranged from 3.13 to 6 months for individual children.

### Statistical analysis

Measures of PAH–DNA adducts and birth outcomes including birth weight, birth length, and head circumference were log-transformed to normalize the distribution and stabilize the variance. The paired Student’s *t*-test was used to compare the maternal and cord blood adduct levels. In testing associations with health outcomes, cord blood adducts were used as the independent variable as in prior studies (Perera et al. 1998, 2004). We used multiple linear regression to analyze the association between PAH–DNA adduct level and either birth outcomes or child development, adjusting for potential confounders. Environmental tobacco smoke (ETS), sex, maternal height, and maternal weight were associated with one or more outcomes (*p* < 0.1) and were considered to be potential confounders of the association between PAH–DNA adducts and birth outcomes and children’s physical growth. Dietary PAH was not included as a covariate because it did not significantly contribute to the final models. High adducts were defined as greater than the median of the detectable adduct value, or 0.36 adducts/10^−8^ nucleotides.

Outcomes analyzed in this study included birth weight, length, and head circumference, as well as child weight, length, and head circumference at 18, 24, and 30 months of age. Gestational age was found to be a significant predictor of birth outcomes and was therefore added to the models involving birth outcome analysis. In all analyses involving head circumference, maternal head circumference and cesarean status were also considered as covariates. Because analyses of physical development involved repeated measurements, we used linear mixed models with random subject effect to account for the within-subject correlation (Littell 1996).

## Results

The demographic characteristics of the 150 mother–newborn pairs from this cohort study are shown in Table 1. The mean cord blood adduct level (0.33 ± 0.14 adducts/10^8^ nucleotides) was somewhat higher than the maternal adduct level (0.29 ± 0.13 adducts/10^8^ nucleotides). Although the difference was not significant, given the estimated 10-fold lower dose to the fetus compared with the mother, the comparable levels of adducts provide evidence that human fetuses are more susceptible than adults to the genotoxic effects of PAHs (Perera et al. 2005).

High cord blood adduct level was significantly associated with decreased birth head circumference (*p* = 0.057) and reduced infant/child weight at 18 months (*p* = 0.03), 24 months (*p* = 0.027), and 30 months of age (*p* = 0.049) (see Table 2). However, maternal adduct level was neither significantly correlated with cord blood adduct level (*r* = 0.140, *p* = 0.299) nor significantly associated with fetal and child growth.

The interaction term between sex and cord blood PAH–DNA adduct level was not significant, suggesting that the strength of association between adduct level and fetal and child growth was not significantly different between females and males. Because higher power is required to detect significance in the interaction term, the lack of significance of the sex × adduct interaction term was likely due to the lower power from the study’s small sample size. However, significant associations between higher cord blood adduct level and either adverse birth outcome or worse physical development were found only among female infants. Among females, high cord blood adduct level was significantly associated with smaller birth head circumference (*p* = 0.022), as well as lower weight at 18 months (*p* = 0.014), 24 months (*p* = 0.012), and 30 months of age (*p* = 0.033) and shorter length at 18 months of age (*p* = 0.033). Among male infants, the corresponding associations were inverse but not significant.

There was a significant association between longer duration of exposure and shorter length at birth (*p* = 0.032) and height at 18 months (*p* = 0.001), 24 months (*p* < 0.001), and 30 months of age (*p* < 0.001) (Table 3). Among females, longer duration of exposure was significantly associated with shorter length at 18 months (*p* = 0.024), 24 months (*p* = 0.018), and 30 months (*p* = 0.019), whereas among males, longer duration of exposure was also significantly associated with shorter length at 18 months (*p* = 0.019), 24 months (*p* = 0.003), and 30 months (*p* = 0.001).

In the mixed model evaluating repeat measures of physical growth from birth through age 30 months, high cord blood adduct level was significantly associated with lower weight (*p* = 0.023) and with smaller head circumference (*p* = 0.056) in childhood. Duration of exposure was significantly associated with shorter length (*p* = 0.0001).

We evaluated the association between distance from the power plant and cord adduct level as well as the association between distance and birth outcomes and physical development. Cord adduct level was negatively but not significantly correlated with distance (*r* = −0.115, *p* = 0.190). Longer distance from the power plant was significantly associated with greater birth length (*p* = 0.03), but consistently positive associations between distance and birth outcomes and physical development were not found. Therefore, our analyses indicate that distance is not a good predictor of PAH exposure.

## Discussion

This study examined the associations between cord blood PAH–DNA adducts and birth outcomes and physical growth in a Chinese population exposed to coal-burning emissions. There was a significant association between elevated cord blood adducts and reduced birth head circumference, and with reduced physical growth (decreased weight at 18, 24, and 30 months of age), after adjusting for potential confounders. These are potentially important findings because several previous studies have reported a correlation between reduced fetal growth and poorer cognitive outcomes (Chaikind and Corman 1991; Desch et al. 1990; Matte et al. 2001). The findings are also consistent with prior reports that PAH–DNA cord blood adducts in Caucasian, African-American, and Dominican newborns were significantly associated with reduced fetal growth, alone or in combination with ETS exposure (Perera et al. 1998, 2003).

As noted above, distance from the power plant was not a good predictor of PAH exposure or of outcomes. The present analysis did not include dispersion modeling to take into account meteorologic factors such as wind speed and wind direction. Because of these factors, subjects who live the same distance from the power plant may receive different levels of plant emission exposure, depending on their residential coordinate relative to the plant. This may account for the observation that the distance measurement alone was not a good predictor of adduct level or birth outcomes and physical development. Future analyses will use dispersion modeling to account for the impact of meteorologic factors.

Fetal toxicity from PAHs may be caused by antiestrogenic effects (Bui et al. 1986), binding of constituents to the human aryl hydrocarbon receptor to induce P450 enzymes (Manchester et al. 1987), DNA damage resulting in activation of apoptotic pathways (Meyn 1995; Nicol et al. 1995; Wood and Youle 1995), binding to receptors for placental growth factors resulting in decreased exchange of oxygen and nutrients, or direct effects of carbon monoxide (Dejmek et al. 2000; National Research Council 1986).

In the multiple linear regression analysis, we found that the association between greater adduct level and decreased at-birth head circumference and physical development was significant only among females. In males, there was an inverse relationship between cord blood adduct level and birth outcome/physical development, but the associations were not significant. Although our small sample size provided insufficient power to detect significance in the interaction term, the findings suggest that female fetuses may be more susceptible to the toxicity of PAHs than male fetuses. The mechanism for a possible sex difference is not clear; however, sexual dimorphism of *P450* gene expression may lead to higher susceptibility of female fetuses to the toxicity of PAHs compared with male fetuses. CYP1A1 and CYP1B1 are responsible for bioactivating PAHs such that they covalently bind to DNA to form harmful PAH–DNA adducts (Nebert et al. 2004). According to Finnstrom et al. (2002), the expression of CYP1B1 is significantly higher in leukocytes of women than men, and Lin et al. (2003) showed that CYP1A1 and CYP1B1 levels in noninduced lymphocytes were significantly higher in female nonsmokers than in male nonsmokers. In summary, higher levels of CYP1 have been found in females in various studies, whether in the noninduced state or after induction by a chemical compound, which may result in sex differences in susceptibility to the toxic effects of PAHs and other combustion-related pollutants (Finnstrom et al. 2002; Iba et al. 1999; Lin et al. 2003; Oropeza-Hernandez et al. 2003). Our small sample size provided insufficient power to detect significance in the interaction term for sex as an effect modifier of the association between adduct level and birth outcomes and physical development. Future studies with larger samples are needed to determine whether sex is a significant effect modifier of the association between adduct level and birth outcomes and physical development.

Maternal adduct level was neither significantly correlated with cord blood adduct level nor significantly associated with fetal and child growth. This suggests that the biologic dose received by the fetus, which depends partly on its genetic profile, is more relevant than the maternal dose to fetal and child growth. Maternal adduct level may not correlate with the cord blood adduct level because of the different genetic profiles of the mother and the child. Our previous study involving Polish mothers and newborns found that *CYP1A1* polymorphisms were not associated with maternal adduct level but were associated with umbilical WBC adduct level (Whyatt et al. 1998).

We also found that fetal and child growth were affected differently by PAH–DNA adduct level and duration of exposure. Higher adduct level was significantly associated with decreased birth head circumference and reduced weight at 18, 24, and 30 months of age, whereas longer duration of exposure was associated with reduced birth length and shorter height at 18, 24, and 30 months of age. In addition, although significant associations between adduct level and fetal and child growth were only found among females, significant associations between duration of exposure and fetal and child growth were present among both males and females. A possible explanation of why longer duration of exposure is significantly associated with worse growth outcomes in both sexes, not just among females, is that although adducts represent the amount of exposure to BaP, duration of exposure represents the amount of exposure to multiple harmful compounds released by the power plant, including other PAHs, PM, and metals. As mentioned above, we were able to attribute seasonal variation in air pollution largely to power plant emissions, which supports the assumption that a longer period of overlap between gestation and plant operation is a proxy for higher *in utero* exposure to ambient PAHs and PM_2.5_ and other coal-burning emissions.

This study has the advantage of being based on individual data as well as medical record and questionnaire data. In addition, we were able to quantitatively measure the individual biologically effective dose of BaP through the measurement of BaP–DNA adducts. However, the study was limited by the modest number of subjects (150) for whom data from all relevant domains were available. Because most study subjects were exposed to power plant emission in both the second and third trimesters, we could not evaluate trimester-specific effects of exposure on fetal and child growth. Another limitation was that children received postnatal exposure to power plant emission in addition to prenatal exposure. Because of the power needs of the Tongliang community, the power plant was shut down later than anticipated, and subjects continued to receive exposure to the plant emission after birth. It is therefore not possible to separate the impact of postnatal exposure from that of prenatal exposure on child physical development.

Results from phase 2 of the Tongliang project, which is studying a second cohort of children who were conceived after the power plant shutdown and did not have *in utero* exposure to power plant emissions, will be presented in future publications.

In conclusion, these results indicate that PAHs from coal-burning power plants are harmful to the developing fetus and child and have implications for energy policy and health. The Tongliang coal-burning power plant was shut down without serious economic consequences to the city, and the energy needs formerly met by the plant were subsequently provided by power from the grid. Because coal-fired power plants currently produce 75% of China’s electricity and most new plants are being built to burn coal, results from the Tongliang study have implications for the health of many other children in China.

## Figures and Tables

**Table 1:** Demographic characteristics of the population (*n* = 150).

Characteristic	Values
Maternal age (years)	25.3 ± 3.2
Maternal education (%)	
< High school	48.3
≥ High school	51.7
Maternal ETS (hr/day)	0.42 ± 1.19
Maternal height (cm)	157.9 ± 3.8
Maternal prepregnancy weight (kg)	49.6 ± 5.8
Maternal head circumference (cm)	54.5 ± 1.3
Cesarean delivery (%)	54
Gestational age (days)	277.8 ± 10.9
Newborn birth weight (g)	3337.5 ± 388.1
Newborn birth length (cm)	50.3 ± 1.7
Newborn head circumference (cm)	33.8 ± 1.1
Sex of newborn (% female)	49.7

Values are mean ± SD or percent.

**Table 2:** Association between cord blood PAH–DNA adducts (dichotomized high/low) and birth outcomes/physical growth.^a^

	Birth		18 months		24 months		30 months	
	β(*n*)	*p*-Value	β(*n*)	*p*-Value	β(*n*)	*p*-Value	β(*n*)	*p*-Value
Weight	−0.007 (112)	0.738	−0.048 (110)	0.03	−0.041 (118)	0.027	−0.040 (119)	0.049
Length or height	−0.001 (112)	0.89	−0.005 (110)	0.483	−0.007 (118)	0.281	−0.006 (119)	0.437
Head circumference	−0.011 (112)	0.057	−0.012 (109)	0.085	−0.006 (118)	0.188	−0.005 (118)	0.311

a Models included ETS, sex, maternal height, and maternal weight as covariates. Gestational age was additionally considered as a covariate for birth outcome analysis, and maternal head circumference and cesarean status were additionally considered as covariates for all analyses involving head circumference.

**Table 3:** Association between duration of exposure and birth outcomes/physical growth.^a^

	Birth		18 months		24 months		30 months	
	β(*n*)	*p*-Value	β(*n*)	*p*-Value	β(*n*)	*p*-Value	β(*n*)	*p*-Value
Weight	−0.010 (128)	0.345	−0.021 (126)	0.078	−0.015 (133)	0.117	−0.002 (135)	0.846
Length or height	−0.007 (128)	0.033	−0.011 (126)	0.001	−0.012 (133)	< 0.001	−0.014 (135)	< 0.001
Head circumference	0.002 (128)	0.618	−0.003 (125)	0.452	−0.004 (133)	0.078	−0.002 (134)	0.529

a Models included ETS, sex, maternal height, and maternal weight as covariates. Gestational age was additionally considered as a covariate for birth outcome analysis, and maternal head circumference and cesarean status were additionally considered as covariates for all analyses involving head circumference.
